# Morphological Changes in Cortical and Subcortical Structures in Multiple System Atrophy Patients With Mild Cognitive Impairment

**DOI:** 10.3389/fnhum.2021.649051

**Published:** 2021-03-23

**Authors:** Chenghao Cao, Qi Wang, Hongmei Yu, Huaguang Yang, Yingmei Li, Miaoran Guo, Huaibi Huo, Guoguang Fan

**Affiliations:** ^1^Department of Radiology, The First Hospital, China Medical University, Shenyang, China; ^2^Department of Radiology, Liaoning Thtombus Treatment Center of Integrated Chinese and Western Medicine, Shenyang, China; ^3^Department of Neurology, The First Hospital, China Medical University, Shenyang, China

**Keywords:** multiple system atrophy, mild cognitive impairment, magnetic resonance imaging, morphometric alterations, FreeSurfer software

## Abstract

**Objective:**

This study aimed to investigate the morphometric alterations in the cortical and subcortical structures in multiple system atrophy (MSA) patients with mild cognitive impairment (MCI), and to explore the association with cognitive deficits.

**Methods:**

A total of 45 MSA patients (25 MSA-only, 20 MSA-MCI) and 29 healthy controls were recruited. FreeSurfer software was used to analyze cortical thickness, and voxel-based morphometry was used to analyze the gray matter volumes. Cortical thickness and gray matter volume changes were correlated with cognitive scores.

**Results:**

Compared to healthy controls, both MSA subgroups exhibited widespread morphology alterations of brain structures in the fronto-temporal regions. Direct comparison of MSA-MCI and MSA-only patients showed volume reduction in the left superior and middle temporal gyrus, while cortical thinning was found in the left middle and inferior temporal gyrus in MSA-MCI patients. Cortical thinning in the left middle temporal gyrus correlated with cognitive assessment and disease duration.

**Conclusion:**

Structural changes in the brain occur in MSA-MCI patients. The alteration of brain structure in the left temporal regions might be a biomarker of cognitive decline in MSA-MCI patients.

## Introduction

Multiple system atrophy (MSA) is a progressive neuro-degenerative disease with a wide range of motor and non-motor symptoms. Current research, assessments, and interventions mainly focus on motor impairment in patients with MSA, such as gait ataxia, limb ataxia, and scanning dysarthria (Iodice et al., 2012). However, relatively little research has been done on non-motor symptoms. As a domain that cannot be ignored in non-motor symptoms, cognitive impairment (CI) affects the processing speed and executive functions of MSA patients, and brings great burden to patients and their families (Koga et al., 2017). Unfortunately, the neuropathological mechanisms of cognitive impairment in MSA patients remain unclear, and this uncertainty might limit further research on MSA patients with cognitive deficits.

Structural magnetic resonance imaging (MRI) is a powerful tool for the study of neuro-degenerative disease. Previous structural MRI studies mainly used voxel-based morphometry (VBM) measures to investigate gray matter (GM) abnormalities. Significant subcortical structural impairment was found in MSA patients with CI (Lee et al., 2016). Further studies found that GM atrophy in the bilateral thalamus, the left cerebellum, and the left pericalcarine gyrus was significantly associated with cognitive deficits in the cerebellar subtype of MSA (Lee et al., 2016); In the parkinsonian subtype of MSA, cerebellum, striatal, temporal, and frontal cortical areas of GM atrophy showed correlation with cognitive deficits (Kim et al., 2015; Caso et al., 2019). These results illustrated that cognitive dysfunction was associated with widespread fronto-temporal subcortical GM, basal ganglia, and cerebellum atrophies in MSA patients (Fiorenzato et al., 2017). These studies focused on subcortical and infratentorial structural alterations in MSA patients, while little attention was paid to the changes of the cortex. Primary cortical injury and subcortical structural degeneration were reported to co-exist in MSA patients (Kim et al., 2013). Previous studies confirmed that the injury of motor cortex in MSA patients contributed to motor dysfunction (Su et al., 2001), but unfortunately studies aimed at the changes of cortical morphology in MSA patients with mild cognitive impairment (MCI) were lacking.

FreeSurfer is a software suite utilizing surface-based morphological analysis that provides accurate information on cortical thickness and is considered the gold standard for measuring cortical thickness (Seiger et al., 2018). Colloby et al. (2019) used FreeSurfer to explore dementia with Lewy bodies and Alzheimer’s disease (AD) dementia and found that, compared to AD, dementia with Lewy bodies retained the cortical structure of the medial temporal lobule, which could be a biomarker for dementia with Lewy bodies. Hanford et al. (2019), in a FreeSurfer study of schizophrenia, found a significant relationship between the cognitive impairment and the cortical thickness of the prefrontal cortex. These studies demonstrate the great potential of FreeSurfer in cortical studies in MSA-MCI patients.

The purpose of our study was to use FreeSurfer analysis combined with VBM to investigate the brain morphological changes that might cause cognitive decline in MSA-MCI patients. The results might help to better understand the relationship between CI and brain structure in MSA patients. We hypothesized that the changes in brain structure, particularly in the fronto-temporal lobules, were associated with the cognitive decline in MSA-MCI patients.

## Materials and Methods

### Subjects

Forty-five patients [25 MSA-no cognitive impairment (NCI) and 20 MSA-MCI patients] from the neurology outpatient clinic at the First Affiliated Hospital of China Medical University were enroled. Patients were diagnosed by a movement disorder specialist with “probable MSA” according to the MSA diagnostic criteria (second edition, 2008) (Gilman et al., 2008). Further, we recruited 29 age-, gender-, and education-matched healthy controls (HC). This study was approved by the ethics review committee of the China Medical University. Patient’s informed consent was obtained before the experiment. All participants had no mental or depressive symptoms. All participants were right-handed, Han Chinese, and had no contraindications to MRI. Subjects were excluded if they had: (i) symptomatic onset before 30 years of age, (ii) substance abuse that could cause cognitive impairment, or (iii) history of neurological illness.

### Clinical and Neuropsychological Assessment

Cognitive status was assessed using the Montreal Cognitive Assessment (MoCA). We used the Movement Disorders Society Task Force criteria to assess MCI (Goldman et al., 2014). For the level I criteria, we defined cognitive impairment as an MoCA score < 26. For the level II criteria, we used 1.5 standard deviations (SD) below the normative values (healthy controls) as a cutoff. Patients were excluded if they fulfiled the Task Force criteria for Parkinson’s disease dementia: (i) MMSE score ≤ 25, (ii) cognitive deficiency severe enough to impair daily life, and (iii) impairment in more than one cognitive domain. Healthy controls with an MoCA score < 26, or < 25 for secondary school-educated subjects (12 years of education), were excluded. Additionally, parts II and III of the Unified Multiple System Atrophy Rating Scale (UMSARS) were used to rate the disease severity.

### MRI Acquisition

MRIs were acquired on a 3.0T MRI scanner (Magnetom Verio, Siemens Healthineers, Germany) equipped with a 32-channel head coil located at the First Affiliated Hospital of China Medical University. High-resolution three-dimensional sagittal T1-weighted images were acquired in a magnetization-prepared rapid acquisition gradient-echo sequence with the following parameters: TR, 5,000 ms; TE, 2,960 ms; FOV, 256 × 256 mm^2^; flip angle, 12°; dist. Factor, 0.5; matrix size, 256 × 256; slice thickness, 1 mm; voxel size, 1.0 × 1.0 × 1.0 mm; slice number, 176.

### Imaging Data Preprocessing

#### Cortical Thickness Measurement

FreeSurfer image analysis suite, version 6.0 (Fischl and Dale, 2000)^1^, was used for cortical reconstruction and thickness analysis on the three-dimensional T1-weighted images data. Details of the methodological have been described previously (Fischl et al., 1999). In brief, the analysis pipeline included non-uniform intensity correction; Talairach transformation; removal of non-brain tissue; segmentation of the images into GM, white matter (WM), and cerebrospinal fluid (CSF); and tessellation of WM/GM boundary. The surface was then wrapped following intensity gradients to optimally identify the WM/GM and GM/CSF (pial) borders. In this segmentation procedure, we can manually edit by adding control points if necessary. Surface inflation, spherical atlas registration, and the parcelation of the cortex into gyral and sulcal structures were performed. Cortical thickness was then defined as the shortest distance from WM/GM boundary to pial surface at each vertex. Finally, the surface maps were realigned to a common surface space and smoothed with a 10 mm full width at half maximum surface-based Gaussian kernel.

### Whole-Brain VBM Analysis

VBM was performed using SPM12 software package (Ashburner and Friston, 2000)^2^. The Diffeomorphic Anatomical Registration Through Exponentiated Lie Algebra (DARTEL) registration method (Ashburner, 2007) was used to analyze whole-brain GM abnormalities. All three-dimensional T1-weighted images were segmented into GM, WM, and CSF in MNI space. The study-specific template was created by DARTEL procedure, and the initial registration of the template into the tissue probability map was performed to create wrapped images. The Jacobian determinant was used for modulation to guarantee that the relative volume of GM was retained after the spatial normalization. The resulting images were smoothed with an 8 mm full width at half maximum Gaussian kernel.

### Statistical Analysis

SPSS 20.0 software (IBM Software Analytics, New York, NY) was used to perform demographic, clinical, and cognitive data analysis. The Q-Q plot and the Kolmogorov-Smirnov test were used to check normal distribution assumption of the variables. The chi-square test, the two-sample *t*-test, the Kruskal-Wallis test, and the Mann-Whitney test were performed for pairwise comparisons of the variables. All the statistically significant thresholds were set at 0.05.

Statistical analysis of cortical thickness was performed using FreeSurfer software. A general linear model was used to explore the differences of cortical thickness between the MSA subgroups and the HC group. The cluster-level statistical threshold was set at *p* < 0.05 and false discovery rate (FDR) method for multiple comparisons was used with age, gender, education, and disease duration as covariates of no interest. If no results were identified through this correction, a less strict threshold of *p* < 0.01, Monte Carlo corrected, was performed. The whole-brain GM volumes obtained with SPM software were compared among MSA subgroups and the HC group, and *post hoc* analysis was performed to explore between-group GM volume differences with the total intracranial volume, age, gender, and education added as covariates. Multiple comparisons were performed using the family wise error (FWE) correction, and the cluster-level statistical threshold was set at *p* < 0.05. Considering that our data do not conform to a normal distribution, Spearman correlation analysis (SPSS 20.0) and the regression models (SPM12) were performed to investigate the correlation of cortical thickness measures and GM volumes with the clinical and cognitive variables. A significance threshold of *p* < 0.05 was set.

## Results

### Demographic, Clinical, and Cognitive Findings

Forty-five MSA patients (MSA-NCI, 25; MSA-MCI, 20) and 29 HCs were enroled. There were no significant differences in age, sex, or level of education between groups. Both patient groups showed significantly lower MoCA scores compared with the HC group, while MSA-MCI patients showed significantly lower MoCA scores and higher UMSARSII scores compared with MSA-NCI patients, as expected. There were no significant differences between MSA-MCI and MSA-NCI patients in terms of UMSARSIII scores and duration of illness (Table 1).

**TABLE 1:** Demographic, clinical, and cognitive data of MSA and HC.

Demographic variable	MSA-NCI (*n* = 25)	MSA-MCI (*n* = 20)	HC (*n* = 29)	Z/χ2	*p*-value
Age (years)	62.52 ± 6.90	64.15 ± 9.22	61.17 ± 6.77	0.672	0.715
Sex (F:M)	12: 13	14: 6	20: 9	3.225	0.199
Education (years)	11.32 ± 3.18	11.70 ± 2.97	12.52 ± 3.10	2.040	0.361
Disease duration (years)	4.44 ± 2.10	3.80 ± 2.19	–	–1.254	0.210
MoCA	26.80 ± 1.22	20.10 ± 2.71	27.17 ± 1.23	46.454	0.000*
UMSARSII	7.24 ± 5.16	15.60 ± 2.76	–	4.592	0.000*
UMSARSIII	34.00 ± 13.59	38.90 ± 17.48	–	1.006	0.314

Variables are mean ± standard deviation: MSA, multiple system atrophy; MCI, mild cognitive impairment; NCI, no cognitive impairment; HC, healthy controls; MoCA, Montreal Cognitive Assessment; UMSARSII and UMSARSIII, Unified Multiple System Atrophy Rating Scale parts II and part III; p < 0.05 was considered statistically significant; 0.000*, values < 0.000.

### Cortical Thickness

**FIGURE 1:**
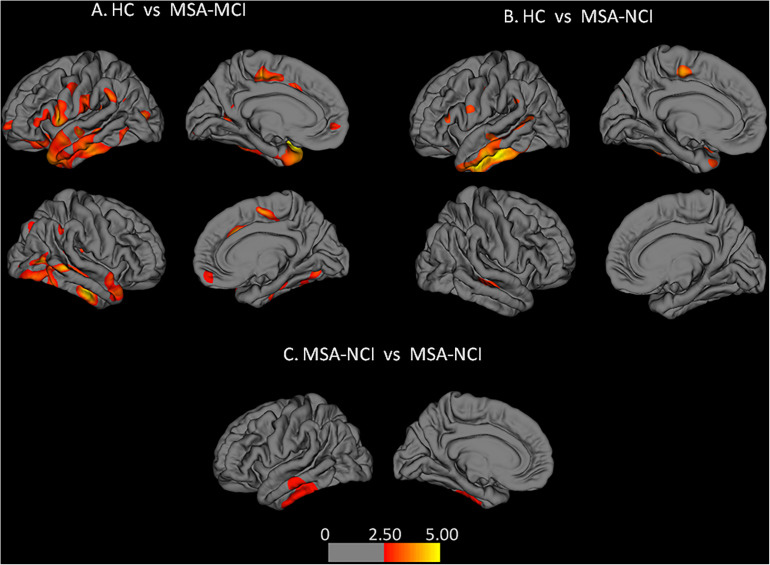
Alterations of cortical thickness: **(A,B)** false discovery rates correction was used (*p* < 0.05); **(C)** left hemisphere after Monte Carlo correction (*p* < 0.01). Warm colors represent areas of cortical thinning. Color bar represents *t*-values.

Compared with the HC group, MSA-MCI patients showed significant cortical thinning in the frontal and temporal cortex of both hemispheres, especially in the bilateral superior frontal gyrus, bilateral superior temporal gyrus, bilateral medial orbito-frontal cortex, and supramarginal gyrus. Other areas of significant cortical thinning were found in the bilateral inferior parietal lobule, right inferior temporal gyrus and superior parietal lobule, left insular cortex and isthmus of cingulate, left paracentral gyrus, and left inferior frontal gyrus. Moreover, cortical thickness of the right middle temporal gyrus, left inferior and superior temporal gyrus, left paracentral lobule, left fusiform gyrus, left supramarginal gyrus, and pars triangularis were decreased in the MSA-NCI group compared with the HC group (*p* < 0.05 FDR). Compared to MSA-NCI patients, MSA-MCI patients showed cortical thinning in the left middle and inferior temporal gyrus (*p* < 0.01 Monte Carlo corrected) (Figure 1).

### Gray Matter Volumetry

**FIGURE 2:**
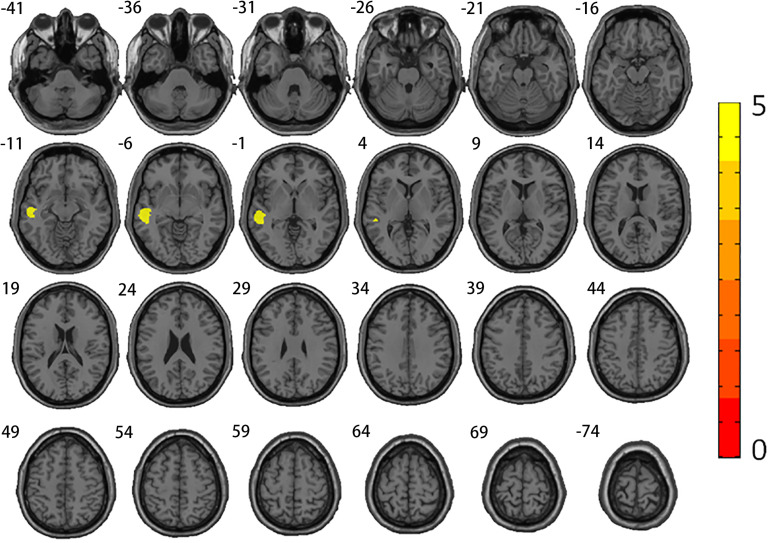
Alterations of gray matter volumes. Comparison between MSA-MCI patients and MSA-NCI patients. Family-wise error correction was used (*p* < 0.05). Warm colors represent areas of GM atrophy.

Regarding GM differences, MSA-MCI patients showed significantly reduced GM in the bilateral cerebellum, bilateral temporal area, right parahippocampal gyrus, left cingulate, and left putamen compared with the HC group. Relative to the HC group, MSA-NCI patients showed significantly decreased GM in the bilateral cerebellum, right fusiform gyrus extending to parahippocampal gyrus areas, and left lingual gyrus extending to fusiform gyrus areas. Compared with the MSA-NCI groups, MSA-MCI patients lost GM volume in the left middle temporal gyrus extending to superior temporal gyrus (*p* < 0.05 FWE) (Table 2 and Figure 2).

**TABLE 2:** Brain regions with significant GM volume reduction between MSA subgroups and HC.

Index brain region	Cluster size	Peak MNI coordinates			*T*-values	*Z*-score	*P*-value
	(Voxels)	X	Y	Z			
**HC vs. MSA-MCI**							
Temporal_Inf_L	871	–34.5	–3	–42	7.67	6.06	0.000*
Fusiform_R	1,544	36	–19.5	–31.5	8.08	6.27	0.000*
Amygdala_R	715	18	−**1**.5	–13.5	6.22	5.23	0.000*
Temporal_Mid_R	48	60	–27	–12	5.77	5.29	0.002
Putamen_L	457	–19.5	10.5	–12	6.40	5.34	0.000*
Cingulum_Mid_L	31	–4.5	–18	43.5	5.79	4.96	0.011
Temporal_Mid_L	59	–52.5	–55.5	12	6.32	4.94	0.002
**HC vs. MSA-NCI**							
Fusiform_R	913	37.5	–21	–30	4.53	4.12	0.015
Lingual_L	1,894	–18	–61.5	–12	4.73	4.27	0.002
**MSA-NCI vs. MSA-MCI**							
Temporal_Mid_L	1,237	–58.5	–25.5	–6	5.39	4.63	0.001

Clusters of GM identified by SPM analysis (p < 0.05, FWE; threshold K > 20 voxels) L, R: left and right; MNI coordinates: Montreal Neurological Institute coordinates; P-value: cluster level, FWE correct; 0.000*, values < 0.000.

### Correlations Between Cognitive Deficits and MRI Findings

**FIGURE 3:**
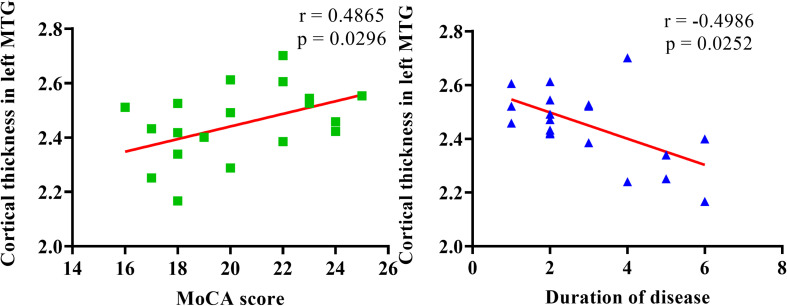
Scatter plots of cortical thickness and MoCA scores/duration of disease in left MTG.

In MSA-MCI patients, UMSARSII scores showed significant negative correlation with the extent of cortical thinning in the left precentral gyrus. Significant positive correlations between GM volume and MoCA scores were observed in the right superior and middle frontal gyrus. However, cortical thickness in the left middle temporal gyrus showed significant positive correlation with MoCA scores and negative correlation with duration of disease. No correlations were found with other clinical and cognitive deficits (Figure 3).

## Discussion

To explore the contribution of whole-brain morphology alterations on CI in MSA-MCI patients, based on the results of FreeSurfer and VBM, we investigated the relationship between cortical and subcortical structures and clinical measurements. Compared to the HC group, MSA-MCI patients showed widespread structural alterations in the fronto-parieto-temporal lobule and the limbic system. In the direct comparison between MSA-MCI and MSA-NCI, we found that MSA-MCI patients showed extensive GM atrophy in left superior and middle temporal gyrus and cortical thinning in left middle and inferior temporal gyrus. We also observed that cortical thinning of the left middle temporal gyrus was associated with cognitive decline and disease duration in MSA-MCI patients, which was in line with our hypotheses.

Several previous studies of a large sample of patients have confirmed the presence of cognitive impairment in MSA patients (Koga et al., 2017). Our results suggested that the alterations in cortical and subcortical structures in the left temporal region were a major cause of cognitive decline in MSA-MCI patients. Similar results have been reported in previous cross-sectional (Donix et al., 2018) and longitudinal (Whitwell et al., 2008) studies of patients with MCI. These studies demonstrated that the presence of significant temporal atrophy in MCI patients can be used to predict progression to dementia. However, our findings contradicted some previous studies of MSA patients (Chang et al., 2009; Fiorenzato et al., 2017; Koga et al., 2017; Caso et al., 2019), which suggested that morphological alterations in frontal lobule played a key role in cognitive impairment in MSA patients. Koga et al. (2017) and Caso et al. (2019) found that cognitive declines emanated from impaired frontal lobule in MSA. Fiorenzato et al. (2017) divided MSA patients into two groups, normal cognitive function and cognitive impairment, and they found that MSA patients with cognitive impairment showed greater frontal atrophy, illustrating that the dysfunction of the frontal-striatal connections might lead to cognitive impairment in MSA patients. Armstrong et al. (2005) found significant hypoperfusion in the frontal lobes of MSA patients, and hypoperfusion in the dorsolateral prefrontal cortex was associated with the severity of cognitive impairment. Both results were confirmed by anatomic and postmortem pathology studies, suggesting that the loss of neurons, astrocytic proliferation, and cytoplasmic inclusion bodies in the fronto-temporal regions might explain the cognitive decline in some MSA patients (Barcelos et al., 2018). Compared with these studies, the patients we enroled in our study were confined to the range of MCI, which might be a reason for the inconsistency between the results of our study and previous studies.

The temporal lobule plays a key role in cognition. Previous functional MRI studies suggested that the left temporal regions were involved in many cognitive processes, including semantic aspects of comprehension (superior temporal gyrus) (Friederici et al., 2003), language processing (superior and middle temporal gyrus) (Fan et al., 2017), semantic memory processing (middle temporal gyrus) (Kiehl et al., 2004; Wei et al., 2012; Fan et al., 2017), delayed memory (inferior temporal gyrus) (Kuroki et al., 2006), and visual processing (inferior temporal gyrus) (Kiernan, 2012; Ju et al., 2020). In addition, several recent studies confirmed that MSA-MCI patients had significant language decline, visual function decline, and memory impairment (Caso et al., 2019; Wang et al., 2019). Therefore, morphological changes in the left temporal region might be involved in cognitive impairment in MSA-MCI patients by affecting language, vision, and memory function. Our conjecture was confirmed by functional MRI studies. Zheng et al. (2019) found that the functional connection between cerebellum and left superior temporal gyrus in MSA patients was damaged, resulting in incomplete default mode network structure and multifunctional cognitive impairment. Ren et al. (2018) found that middle temporal gyrus involvement in the cerebellum and temporal cortical circuit functional connection damage caused the memory decline of MSA patients. Our findings were consistent with a previous study (Caso et al., 2019) showing that, for MSA patients, the involvement of not only the frontal but also the temporal regions was associated with cognitive impairment, as detected by structural imaging. These studies all emphasized the critical role of the temporal lobule in cognitive decline in MSA patients. Our findings, supported by Zhang et al. (2018), were consistent with one of the hypotheses regarding the underlying mechanism of cognitive impairment in MSA patients. Since we detected morphological changes in both VBM and FreeSurfer, we believed that the damage to the cerebral cortex and subcortical structure together led to cognitive impairment in MSA patients. Additionally, we also found that the temporal cortical thickness was significantly correlated with disease duration. Thus, the cortical thinning in the temporal region might be a predictor of increased risk for future dementia in MSA patients (Teipel et al., 2010).

Compared with the HC group, MSA-MCI patients also showed more widespread cortical thinning in the frontal lobule, however correlation results showed that the cortical thinning in the frontal lobule had no significant correlation with MoCA scores (*r* = 0.41; *p* = 0.07). Meanwhile no alterations were found in the frontal lobule in the direct comparison between MSA-MCI and MSA-NCI patients. Previous studies (Ozawa et al., 2004; Koga et al., 2017; Caso et al., 2019) have shown significant frontal lobe changes in MSA patients with cognitive impairment. Compared to these studies, the patients in our study were roughly in the mid-stage of disease (mean duration: MSA-MCI, 4.44 years; MSA-NCI, 3.80 years) and the cognitive impairment was relatively mild, so the contradictory results might be due to the disparate course of disease and different cognitive states. Compared with the HC group, we also found more widespread structural alterations in the basal ganglia and limbic system. Not surprisingly, pathologic study demonstrated that severe neuronal cytoplasmic inclusions in the limbic regions such as the hippocampus, the parahippocampal gyrus, the thalamus cortex, and the orbito-frontal cortex were associated with cognitive impairment in MSA patients (Wang et al., 2019).

Of note, the structural alterations in our MSA-MCI patients were mainly in the left rather than right hemisphere, and these results were observed in previous studies (Karas et al., 2003; Yang et al., 2019; Zhou et al., 2020). One possible explanation for this finding was that in our patient group, 33 had right-onset disease and 12 had left-onset disease. Previous reports suggested that ipsilateral brain structural alterations in MSA patients were related to contralateral clinical symptoms, showing significant asymmetry of clinical symptoms (Hwang et al., 2015; Yang et al., 2019). Some studies also suggested that a “time lag” may occur in the development of structural damage in the right hemisphere (Karas et al., 2003; Oishi et al., 2018). However, these conclusions were preliminary and need to be confirmed by more targeted study designs.

The present study has the following limitations. First, our sample size of patients with this rare condition is relatively small. Therefore, some of the differences we found in the regions between MSA-MCI and MSA-NCI did not exist after multiple comparison correction. Second, our study is a preliminary study on the cognitive impairment and morphological alterations of MSA-MCI patients. We did not examine the different effects of the brain on domain-specific cognitive changes. Future studies should focus on the relationship between various cognitive domains and brain structural alterations. Finally, we noticed that autonomic dysfunction might have an impact on the cognitive assessment in our study, so a rough assessment of autonomic function was achieved using UMSARSIII scales. However, more comprehensive and systematic quantitative tools, such as the Scopa-Aut questionnaire, would be helpful for future research.

In conclusion, the present study suggests that cognitive decline due to temporal region alterations might be characteristic of MSA-MCI patients. Since the underlying mechanism and brain alterations of MSA-MCI patients are largely unknown, the results of our study may be helpful to improve the knowledge of health care professionals on the important role of the temporal lobe in the progression of this disease, and this finding may be helpful to the future study of MSA. Future studies need to conduct multiparametric MRI studies on sufficient sample of patients to better understand the brain alterations in MSA patients.

## Data Availability Statement

The raw data supporting the conclusions of this article will be made available by the authors, without undue reservation.

## Ethics Statement

The studies involving human participants were reviewed and approved by The First Affiliated Hospital of China Medical University. The patients/participants provided their written informed consent to participate in this study.

## Author Contributions

CC, QW, and GF contributed to the study conception and design. HYu revised important intellectual content. CC and QW were analyzed and interpreted the data. CC was drafted the manuscript. GF was critically revised the manuscript and involved in study design, data interpretation, manuscript revision, and final approval of the version to be submitted. HYa, MG, YL, and HH performed data acquisition. All authors contributed to the article and approved the submitted version.

## Conflict of Interest

The authors declare that the research was conducted in the absence of any commercial or financial relationships that could be construed as a potential conflict of interest.

## Footnotes

1 http://surfer.nmr.mgh.harvard.edu/

2 http://www.fil.ion.ucl.ac.uk/spm

## References

[B1] Armstrong R. Lantos P. L. Cairns N. J. (2005). Multiple system atrophy: laminar distribution of the pathological changes in frontal and temporal neocortex - a study in ten patients. *Clin. Neuropathol.* 24 230–235. 16167547

[B2] Ashburner J. (2007). A fast diffeomorphic image registration algorithm. *Neuroimage* 38 95–113. 10.1016/j.neuroimage.2007.07.007 17761438

[B3] Ashburner J. Friston K. J. (2000). Voxel-based morphometry–the methods. *Neuroimage* 11(6 Pt 1) 805–821. 10.1006/nimg.2000.0582 10860804

[B4] Barcelos L. B. Saad F. Giacominelli C. Saba R. A. de Carvalho Aguiar P. M. Silva S. M. A. (2018). Neuropsychological and clinical heterogeneity of cognitive impairment in patients with multiple system atrophy. *Clin. Neurol. Neurosurg.* 164 121–126. 10.1016/j.clineuro.2017.10.039 29223069

[B5] Caso F. Canu E. Lukic M. Petrovic I. Fontana A. Nikolic I. (2019). Cognitive impairment and structural brain damage in multiple system atrophy-parkinsonian variant. *J. Neurol.* 267 87–94. 10.1007/s00415-019-09555-y 31559533

[B6] Chang C. C. Chang Y. Y. Chang W. N. Lee Y. C. Wang Y. L. Lui C. C. (2009). Cognitive deficits in multiple system atrophy correlate with frontal atrophy and disease duration. *Eur. J. Neurol.* 16 1144–1150. 10.1111/j.1468-1331.2009.02661.x 19486137

[B7] Colloby S. J. Watson R. Blamire A. M. O’Brien J. T. Taylor J. P. (2019). Cortical thinning in dementia with lewy bodies and parkinson disease dementia. *Aust. N. Z. J. Psychiatry* 54 633–643. 10.1177/0004867419885165 31696728 PMC7285984

[B8] Donix M. Haussmann R. Helling F. Zweiniger A. Lange J. Werner A. (2018). Cognitive impairment and medial temporal lobe structure in young adults with a depressive episode. *J. Affect. Disord.* 237 112–117. 10.1016/j.jad.2018.05.015 29803901

[B9] Fan J. Zhong M. Gan J. Liu W. Niu C. Liao H. (2017). Spontaneous neural activity in the right superior temporal gyrus and left middle temporal gyrus is associated with insight level in obsessive-compulsive disorder. *J. Affect. Disord.* 207 203–211. 10.1016/j.jad.2016.08.027 27723545

[B10] Fiorenzato E. Weis L. Seppi K. Onofrj M. Cortelli P. Zanigni S. (2017). Brain structural profile of multiple system atrophy patients with cognitive impairment. *J. Neural Transm. (Vienna)* 124 293–302. 10.1007/s00702-016-1636-0 27778099

[B11] Fischl B. Dale A. M. (2000). Measuring the thickness of the human cerebral cortex from magnetic resonance images. *Proc. Natl. Acad. Sci.U.S.A.* 97 11050–11055. 10.1073/pnas.200033797 10984517 PMC27146

[B12] Fischl B. Sereno M. I. Dale A. M. (1999). Cortical surface-based analysis II: Inflation, flattening, and a surface-based coordinate system. *Neuroimage* 9 195–207. 9931269 10.1006/nimg.1998.0396

[B13] Friederici A. D. Shirley-Ann R. Anja H. Fiebach C. J. (2003). The role of left inferior frontal and superior temporal cortex in sentence comprehension: localizing syntactic and semantic processes. *Cereb. Cortex* 13 170–177. 12507948 10.1093/cercor/13.2.170

[B14] Gilman S. Wenning G. K. Low P. A. Brooks D. J. Mathias C. J. Trojanowski J. Q. (2008). Second consensus statement on the diagnosis of multiple system atrophy. *Neurology* 71 670–676. 10.1212/01.wnl.0000324625.00404.15 18725592 PMC2676993

[B15] Goldman J. G. Holden S. Ouyang B. Bernard B. Goetz C. G. Stebbins G. T. (2014). Diagnosing PD−MCI by MDS task force criteria: how many and which neuropsychological tests? *Mov. Disord.* 30 402–406. 10.1002/mds.26084 25449653 PMC4357536

[B16] Hanford L. C. Pinnock F. Hall G. B. Heinrichs R. W. (2019). Cortical thickness correlates of cognitive performance in cognitively-matched individuals with and without schizophrenia. *Brain Cogn.* 132 129–137. 10.1016/j.bandc.2019.04.003 31005042

[B17] Hwang I. Sohn C. H. Kang K. M. Jeon B. S. Kim H. J. Choi S. H. (2015). Differentiation of parkinsonism-predominant multiple system atrophy from Idiopathic parkinson disease using 3T susceptibility-weighted MR imaging. focusing on putaminal change and lesion asymmetry. *Am. J. Neuroradiol.* 36 2227–2234. 10.3174/ajnr.A4442 26338919 PMC7964268

[B18] Iodice V. Lipp A. Ahlskog J. E. Sandroni P. Fealey R. D. Parisi J. E. (2012). Autopsy confirmed multiple system atrophy cases: mayo experience and role of autonomic function tests. *J. Neurol. Neurosurg. Psychiatry* 83 453–459. 10.1136/jnnp-2011-301068 22228725 PMC3454474

[B19] Ju Y. Ou W. Su J. Averill C. L. Liu J. Wang M. (2020). White matter microstructural alterations in posttraumatic stress disorder: an ROI and whole-brain based meta-analysis. *J. Affect. Disord.* 266 655–670. 10.1016/j.jad.2020.01.047 32056942

[B20] Karas G. B. Burton E. J. Rombouts S. A. R. B. van Schijndel R. A. O’Brien J. T. Scheltens P.h, et al. (2003). A comprehensive study of gray matter loss in patients with Alzheimer’s disease using optimized voxel-based morphometry. *NeuroImage* 18 895–907. 10.1016/s1053-8119(03)00041-7 12725765

[B21] Kiehl K. A. Smith A. M. Mendrek A. Forster B. B. Hare R. D. Liddle P. F. (2004). Temporal lobe abnormalities in semantic processing by criminal psychopaths as revealed by functional magnetic resonance imaging. *Psychiatry Res. Neuroimaging* 130 27–42. 10.1016/s0925-4927(03)00106-9 14972366

[B22] Kiernan J. A. (2012). Anatomy of the temporal lobe. *Epilepsy Res. Treat.* 2012:176157. 10.1155/2012/176157 22934160 PMC3420617

[B23] Kim J. S. Yang J.-J. Lee D.-K. Lee J.-M. Youn J. Cho J. W. (2015). Cognitive impairment and its structural correlates in the parkinsonian subtype of multiple system atrophy. *Neurodegener. Dis.* 15 294–300. 26202063 10.1159/000430953

[B24] Kim J. S. Youn J. Yang J. J. Lee D. K. Lee J. M. Kim S. T. (2013). Topographic distribution of cortical thinning in subtypes of multiple system atrophy. *Parkinsonism Relat. Disord.* 19 970–974. 10.1016/j.parkreldis.2013.06.012 23867866

[B25] Koga S. Parks A. Uitti R. J. van Gerpen J. A. Cheshire W. P. Wszolek Z. K. (2017). Profile of cognitive impairment and underlying pathology in multiple system atrophy. *Mov. Disord.* 32 405–413. 10.1002/mds.26874 27859650 PMC5359072

[B26] Kuroki N. Shenton M. E. Salisbury D. F. Hirayasu Y. Onitsuka T. Ersner H. (2006). Middle and inferior temporal gyrus gray matter volume abnormalities in first-episode schizophrenia: an MRI study. *Am. J. Psychiatry* 163 2103–2110. 17151161 10.1176/appi.ajp.163.12.2103 PMC2766919

[B27] Lee M. J. Shin J. H. Seoung J. K. Lee J. H. Yoon U. Oh J. H. (2016). Cognitive impairments associated with morphological changes in cortical and subcortical structures in multiple system atrophy of the cerebellar type. *Eur. J. Neurol.* 23 92–100. 10.1111/ene.12796 26234320

[B28] Oishi A. Yamasaki T. Tsuru A. Minohara M. Tobimatsu S. (2018). Decreased gray matter volume of right inferior parietal lobule is associated with severity of mental disorientation in patients with mild cognitive impairment. *Front. Neurol.* 9:1086. 10.3389/fneur.2018.01086 30619046 PMC6302885

[B29] Ozawa T. Paviour D. Quinn N. P. Josephs K. A. Sangha H. Kilford L. (2004). The spectrum of pathological involvement of the striatonigral and olivopontocerebellar systems in multiple system atrophy: clinicopathological correlations. *Brain* 127(Pt 12) 2657–2671. 10.1093/brain/awh303 15509623

[B30] Ren S. Zhang H. Zheng W. Liu M. Gao F. Wang Z. (2018). Altered functional connectivity of cerebello-cortical circuit in multiple system atrophy (Cerebellar-Type). *Front. Neurosci.* 12:996. 10.3389/fnins.2018.00996 30662394 PMC6328464

[B31] Seiger R. Ganger S. Kranz G. S. Hahn A. Lanzenberger R. (2018). Cortical thickness estimations of freesurfer and the CAT12 toolbox in patients with Alzheimer’s disease and healthy controls. *J. Neuroimaging* 28 515–523. 10.1111/jon.12521 29766613 PMC6174993

[B32] Su M. Yoshida Y. Hirata Y. Watahiki Y. Nagata K. (2001). Primary involvement of the motor area in association with the nigrostriatal pathway in multiple system atrophy: neuropathological and morphometric evaluations. *Acta Neuropathol.* 101 57–64. 10.1007/s004010000273 11194942

[B33] Teipel S. J. Meindl T. Wagner M. Stieltjes B. Reuter S. Hauenstein K.-H. (2010). Longitudinal changes in fiber tract integrity in healthy aging and mild cognitive impairment: a DTI Follow-up study. *J. Alzheimers Dis.* 22 507–522. 20847446 10.3233/JAD-2010-100234

[B34] Wang N. Zhang L. Yang H. Luo X. Fan G. (2019). Do multiple system atrophy and Parkinson’s disease show distinct patterns of volumetric alterations across hippocampal subfields? *An Exploratory Study. Eur. Radiol.* 29 4948–4956. 10.1007/s00330-019-06043-9 30796577

[B35] Wei T. Liang X. He Y. Zang Y. Han Z. Caramazza A. (2012). Predicting conceptual processing capacity from spontaneous neuronal activity of the left middle temporal gyrus. *J. Neurosci.* 32 481–489. 10.1523/JNEUROSCI.1953-11.2012 22238084 PMC6621087

[B36] Whitwell J. L. Shiung M. M. Przybelski S. A. Weigand S. D. Knopman D. S. Boeve B. F. (2008). MRI patterns of atrophy associated with progression to AD in amnestic mild cognitive impairment. *Neurology* 70 512–520. 17898323 10.1212/01.wnl.0000280575.77437.a2 PMC2734138

[B37] Yang H. Wang N. Luo X. Lv H. Liu H. Li Y. (2019). Cerebellar atrophy and its contribution to motor and cognitive performance in multiple system atrophy. *Neuroimage Clin.* 23:101891. 10.1016/j.nicl.2019.101891 31226621 PMC6587071

[B38] Zhang L. Zhang L. Xue F. Yue K. Peng H. Wu Y. N. (2018). Brain morphological alteration and cognitive dysfunction in multiple system atrophy. *Quant. Imaging Med. Surg.* 8 1030–1038. 10.21037/qims.2018.11.02 30598880 PMC6288059

[B39] Zheng W. Ren S. Zhang H. Liu M. Zhang Q. Chen Z. (2019). Spatial patterns of decreased cerebral blood flow and functional connectivity in multiple system atrophy (Cerebellar-Type): a combined arterial spin labeling perfusion and resting state functional magnetic resonance imaging study. *Front. Neurosci.* 13:777. 10.3389/fnins.2019.00777 31417345 PMC6685442

[B40] Zhou Q. H. Wang K. Zhang X. M. Wang L. Liu J. H. (2020). Differential regional brain spontaneous activity in subgroups of mild cognitive impairment. *Front. Hum. Neurosci.* 14:2. 10.3389/fnhum.2020.00002 32082131 PMC7002564

